# Evidence for shear-mediated Ca^2+^ entry through mechanosensitive cation channels in human platelets and a megakaryocytic cell line

**DOI:** 10.1074/jbc.M116.766196

**Published:** 2017-04-17

**Authors:** Zeki Ilkan, Joy R. Wright, Alison H. Goodall, Jonathan M. Gibbins, Chris I. Jones, Martyn P. Mahaut-Smith

**Affiliations:** From the ‡ Department of Molecular and Cell Biology, University of Leicester, Leicester LE1 7RH, United Kingdom,; the § Department of Cardiovascular Sciences, University of Leicester and National Institute for Health Research (NIHR) Cardiovascular Biomedical Research Unit, Glenfield Hospital, Leicester LE3 9QP, United Kingdom, and; the ¶ School of Biological Sciences, University of Reading, Reading RG6 6AS, United Kingdom

**Keywords:** calcium, ion channel, mechanotransduction, platelet, shear stress, Piezo1

## Abstract

The role of mechanosensitive (MS) Ca^2+^-permeable ion channels in platelets is unclear, despite the importance of shear stress in platelet function and life-threatening thrombus formation. We therefore sought to investigate the expression and functional relevance of MS channels in human platelets. The effect of shear stress on Ca^2+^ entry in human platelets and Meg-01 megakaryocytic cells loaded with Fluo-3 was examined by confocal microscopy. Cells were attached to glass coverslips within flow chambers that allowed applications of physiological and pathological shear stress. Arterial shear (1002.6 s^−1^) induced a sustained increase in [Ca^2+^]*_i_* in Meg-01 cells and enhanced the frequency of repetitive Ca^2+^ transients by 80% in platelets. These Ca^2+^ increases were abrogated by the MS channel inhibitor *Grammostola spatulata* mechanotoxin 4 (GsMTx-4) or by chelation of extracellular Ca^2+^. Thrombus formation was studied on collagen-coated surfaces using DiOC_6_-stained platelets. In addition, [Ca^2+^]*_i_* and functional responses of washed platelet suspensions were studied with Fura-2 and light transmission aggregometry, respectively. Thrombus size was reduced 50% by GsMTx-4, independently of P2X1 receptors. In contrast, GsMTx-4 had no effect on collagen-induced aggregation or on Ca^2+^ influx via TRPC6 or Orai1 channels and caused only a minor inhibition of P2X1-dependent Ca^2+^ entry. The Piezo1 agonist, Yoda1, potentiated shear-dependent platelet Ca^2+^ transients by 170%. Piezo1 mRNA transcripts and protein were detected with quantitative RT-PCR and Western blotting, respectively, in both platelets and Meg-01 cells. We conclude that platelets and Meg-01 cells express the MS cation channel Piezo1, which may contribute to Ca^2+^ entry and thrombus formation under arterial shear.

## Introduction

Platelet activation plays a crucial role in the physiological process of hemostasis but is also the key precipitating event leading to arterial thrombosis and thus potentially life threatening pathological events such as myocardial infarction or stroke. In the circulation, shear stress exerted by laminar flow of blood is regarded as a vital environmental factor during platelet activation in both normal and pathological situations. For example, shear stress is required at the early stages of the hemostatic machinery where it unfolds von Willebrand factor to reveal the binding domains to glycoprotein Ib and thus allow attachment to collagen exposed at an injury site (1).

Ion channels have important roles in regulating physiological responses of all cells by controlling transmembrane ionic fluxes. In particular, an increase in [Ca^2+^]*_i_* is a pivotal signaling event that is essential for most major functional responses during platelet activation, including cytoskeletal rearrangements and integrin inside-out signaling (2, 3). Well studied examples of platelet Ca^2+^-permeable ion channels include Orai1 store-operated channels and ATP-gated P2X1 channels (4), which both contribute to arterial thrombosis. In a recent screen of the platelet channelome using quantitative PCR, transcripts for the MS cation channel Piezo1 encoded by the *FAM38A* gene were detected (5). Platelet proteomic and transcriptomic studies also indicate Piezo1 expression in human platelets (6, 7). Piezo1 channels are activated by tension within the lipid bilayer of the membrane itself rather than via a link to the cytoskeleton (8, 9) and have key roles in a range of cellular activities, including erythrocyte volume regulation (10), lineage determination in neural stem cells (11), and vascular development (12). Elucidation of these MS roles for Piezo1 channels have, in part, relied upon pharmacological reagents such as the inhibitor *Grammostola spatulata* mechanotoxin-4, GsMTx-4^3^ from tarantula venom (8, 13, 14), and the recently developed agonist Yoda1 (15, 16).

In the present study, we provide evidence that human platelets and a megakaryocytic cell line express MS Piezo1 ion channels. A novel *in vitro* approach was developed, using PECAM-1 antibodies, to adhere platelets to glass slides without inducing spontaneous activation and thereby permit the study of shear-induced Ca^2+^ responses. Arterial shear stress stimulated GsMTx-4-sensitive Ca^2+^ entry in platelets and Meg-01 cells, providing evidence that they exhibit MS cation channel activity. GsMTx-4 also inhibited thrombus formation under flow, demonstrating a potential role for MS ion channels in platelet function. The stimulation of Ca^2+^ responses by Yoda1 in both Meg-01 cells and platelets together with mRNA and protein expression studies provide evidence that the MS cation channel Piezo1 contributes to the shear-dependent events observed.

## Results

### Intracellular Ca^2+^ responses in Meg-01 cells under shear stress

**Figure 1.**
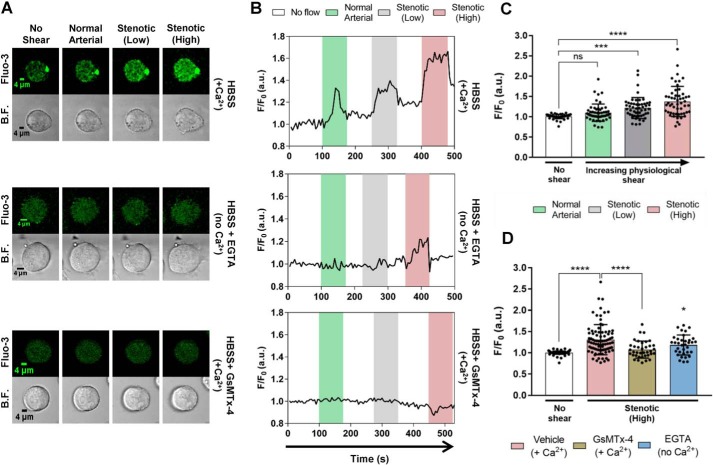
**Fluid shear stress-dependent Ca^2+^ influx in Meg-01 cells is inhibited by GsMTx-4 and chelation of extracellular Ca^2+^.** *A* and *B*, representative images (*A*) and *F*/*F*_0_ fluorescence recordings (*B*) of single Meg-01 cells exposed to arterial and two levels of stenotic shear in HBSS with Ca^2+^, without Ca^2+^ (EGTA), and with GsMTx-4 in the presence of Ca^2+^. *C*, mean peak *F*/*F*_0_ increases (*n* = 53 cells) in response to different shear levels in the presence of extracellular Ca^2+^. *D*, mean peak *F*/*F*_0_ increases under no flow conditions (*n* = 113 cells) and at the high stenotic shear rate with (*n* = 85 cells) and without extracellular Ca^2+^ (*n* = 35 cells) and with GsMTx-4 in the presence of Ca^2+^ (*n* = 37 cells). ****, *p* < 0.0001; *, *p* < 0.05; **, *p* < 0.01. All cells were from cell culture passages 1–11. *B.F.*, bright field.

Meg-01 cells express several platelet lineage surface markers and have been used as a model for studies of signaling in megakaryocytes and platelets (4, 17). We therefore investigated the effect of applied shear stress on [Ca^2+^]*_i_* in this megakaryoblastic cell line as a first step to address our hypothesis that MS cation channels contribute to platelet responses. When Ca^2+^-containing saline was applied at increasing arterial shear rates to Meg-01 cells attached to a glass coverslip, increases in the Fluo-3 signal were observed of a magnitude that correlated with the size of the shear force applied (Fig. 1, *A* and *B*, *top panels*): *F*/*F*_0_ increased from 1.0 ± 0.1 at no shear (0.0 s^−1^) to 1.1 ± 0.2, 1.2 ± 0.3, and 1.4 ± 0.4 at normal arterial (1002.6 s^−1^), stenotic (low) (2282.7 s^−1^), and stenotic (high) (3989.3 s^−1^) shear rates, respectively (Fig. 1*C*). In the absence of extracellular Ca^2+^ (0 Ca^2+^, 1 mm EGTA saline) shear-dependent increases in the Fluo-3 signal were abolished except for a small, residual response (*F*/*F*_0_ = 1.2 ± 0.2) at the highest flow rate (Fig. 1, *A* and *B*, *middle panels*, and *D*). Pretreating the attached Meg-01 cells with 2.5 μm GsMTx-4 abolished all increases in Fluo-3 signal in response to applied shear stress (Fig. 1, *A* and *B*, *bottom panels*, and *D*). We also compared the effect of shear flow in parallel-plate flow chambers on [Ca^2+^]*_i_* in human umbilical vein endothelial cells, which are known to express functional Piezo1 channels (14). Application of arterial shear induced elevations in [Ca^2+^]*_i_* that were abolished by GsMTx-4 as observed in Meg-01 cells.^4^ Increases in [Ca^2+^]*_i_* were also observed when the blunt tip of a glass pipette was used to depress the Meg-01 cell surface, as an alternative mechanical stimulus to shear stress (see Fig. 9). Such glass pipette-induced force has been widely used in the study of Piezo1 channels in HEK 293T cells (9, 15) and a mouse neuroblastoma cell line (18). In the present study, we focused on the use of shear forces applied by fluid flow as a more physiological mechanical stimulus for blood cells.

### Ca^2+^ transients in platelets under shear stress

**Figure 2.**
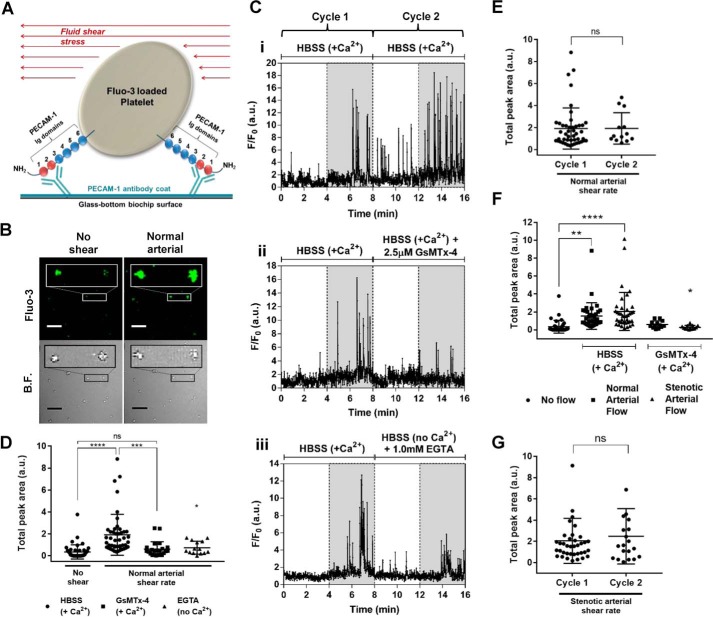
**Fluid shear stress induces Ca^2+^ transients in single platelets that are inhibited by GsMTx-4 and chelation of extracellular Ca^2+^.** *A*, a cartoon representation of single platelet attachment to PECAM-1 antibody-coated biochip surface via the Ig domains 1 and 2 of the platelet PECAM-1. *B*, representative Fluo-3 fluorescence and bright field (*B.F.*) images of individual Fluo-3-loaded attached platelets before and during exposure to arterial shear. *Scale bars*, 20 μm. The *magnified rectangular sections* have been enlarged 3-fold. *C*, representative *F*/*F*_0_ Fluo-3 recordings in single platelets during no applied shear stress (*white regions*) and normal arterial shear (*gray regions*). Two successive cycles of 4 min without shear followed by 4 min of arterial shear were applied, in which the second cycle was used to compare the control conditions (*i.e.* HBSS + Ca^2+^ only) (*panel i*), with the effect of GsMTx-4 (*panel ii*), or removal of extracellular Ca^2+^ (*panel iii*). *D–G*, average Ca^2+^ increases, calculated as the 4-min *F*/*F*_0_ integral of all [Ca^2+^]*_i_* transients. *D*, responses in the absence of shear and during arterial shear, in the presence of extracellular Ca^2+^ with and without GsMTx-4 and in the absence of extracellular Ca^2+^ (*n* = 46, 46, 23, and 14 cells in no shear, HBSS, GsMTx-4, and EGTA, respectively). *, *p* < 0.05, compared with HBSS-only control under shear. *E*, comparison of Ca^2+^ responses during cycles 1 and 2 of normal arterial flow with Ca^2+^-containing HBSS only (*n* = 46 and 13 cells, respectively). No significant difference was found between *F*/*F*_0_ integrals of the calcium transients from cycles 1 and 2. *F*, Ca^2+^ responses in Ca^2+^-containing HBSS in the absence of shear (no applied flow) and during arterial stenotic shear with and without GsMTx-4 (*n* = 36, 36, 38, 13, and 13 cells in no applied flow, HBSS normal arterial, HBSS stenotic arterial, GsMTx-4 normal arterial, and GsMTx-4 stenotic arterial flow conditions, respectively). *, *p* < 0.05, compared with HBSS-only control under stenotic shear. *G*, no significant difference was found between *F*/*F*_0_ integrals of the calcium transients from cycles 1 and 2 of stenotic arterial flow, using Ca^2+^-containing HBSS only (*n* = 38 and 20 cells, respectively). ****, *p* < 0.0001; ***, *p* < 0.001; **, *p* < 0.01. *ns*, not significant.

The shear-induced Ca^2+^ entry observed in Meg-01 cells led us to develop a method to examine whether a similar pathway exists in human platelets. Previous measurements of Ca^2+^ responses under arterial shear in single platelets have used glass coverslips coated with adhesive receptor ligands such as fibrinogen and collagen or synthetic peptides mimicking their binding domains (19–21); however, this approach will generate activation signals including Ca^2+^ mobilization independently of the mechanical stimulus. We therefore used an antibody against the receptor PECAM-1, which is inhibitory to platelet function, to immobilize these cells on glass slides. PECAM-1 normally plays a role in homophilic interactions between Ig domains 1 and 2 of the molecules on nearby platelets (22). PECAM-1 antibody used in this assay (clone WM59) binds to Ig domains 1 and 2, hence inhibiting homophilic binding between platelets. It therefore provides a coat onto which platelets can attach and become immobilized without being activated by the glass surface (23, 24) (Fig. 2*A*). Exposure of attached unstimulated platelets to normal arterial shear stress (1002.6 s^−1^) resulted in multiple transient increases in cytosolic Ca^2+^ after a delay of 1–2 min (Fig. 2, *B* and *C*). Subsequent arrest of flow led to a reduction but not complete inhibition of this Ca^2+^ response, although a second application of arterial shear caused a further increase in the number of Ca^2+^ transients (Fig. 2*C*, *panel i*). The Ca^2+^ responses were quantified using the *F*/*F*_0_ integral for a total of 4 min (*F*/*F*_0_·4 min, in arbitrary units). Prior to the application of flow, when only occasional Ca^2+^ transients were observed, this value was 0.4 ± 0.6; during normal arterial shear it increased significantly to 1.9 ± 1.9 (Fig. 2*D*). In every platelet sample, a proportion of attached platelets did not show increased Ca^2+^ transients in response to arterial shear. However, in the platelets that did respond, the *F*/*F*_0_ integrals during a first and second exposure to either arterial or stenotic shear were not significantly different (Fig. 2, *E* and *G*). This allowed the effects of GsMTx-4 and removal of Ca^2+^ to be assessed during the second cycle of shear. The shear-induced Ca^2+^ transients were significantly reduced by either the addition of 2.5 μm GsMTx-4 (*F*/*F*_0_·4 min value of 0.6 ± 0.6, which is 31.8% of control, *i.e.* 1.9 ± 1.9) or removal of extracellular Ca^2+^ (*F*/*F*_0_·4 min value of 0.7 ± 0.6, which is 38.5% of control) (Fig. 2, *C*, *panels ii* and *iii*, and *D*). A higher arterial shear (3989.3 s^−1^), equivalent to the situation when stenosis or narrowing occurs in the arteries, induced a larger and more significant increase (*F*/*F*_0_·4 min value of 2.1 ± 2.1, *n* = 38, *p* < 0.0001) above prestimulus (no flow) levels when compared with normal arterial shear (Fig. 2*F*) and was also inhibited by GsMTx-4 (*F*/*F*_0_·4 min value of 0.6 ± 0.4, which is 28.6% of control, *i.e.* 2.1 ± 2.1, *n* = 13, *p* < 0.05). Together, these results suggest that platelets, like Meg-01 cells, possess a MS Ca^2+^ influx pathway induced by physiological levels of shear. The major difference between the response in these two cell types was the longer delay between stimulus application and the [Ca^2+^]*_i_* increase in platelets compared with Meg-01 cells. This may result from the greater rigidity of platelet surface membranes, a consequence of the extensive cortical cytoskeleton that will resist deformation and thus activation of MS ion channels (25, 26).

### Effect of GsMTx-4 on thrombus formation and collagen-induced platelet aggregation

**Figure 3.**
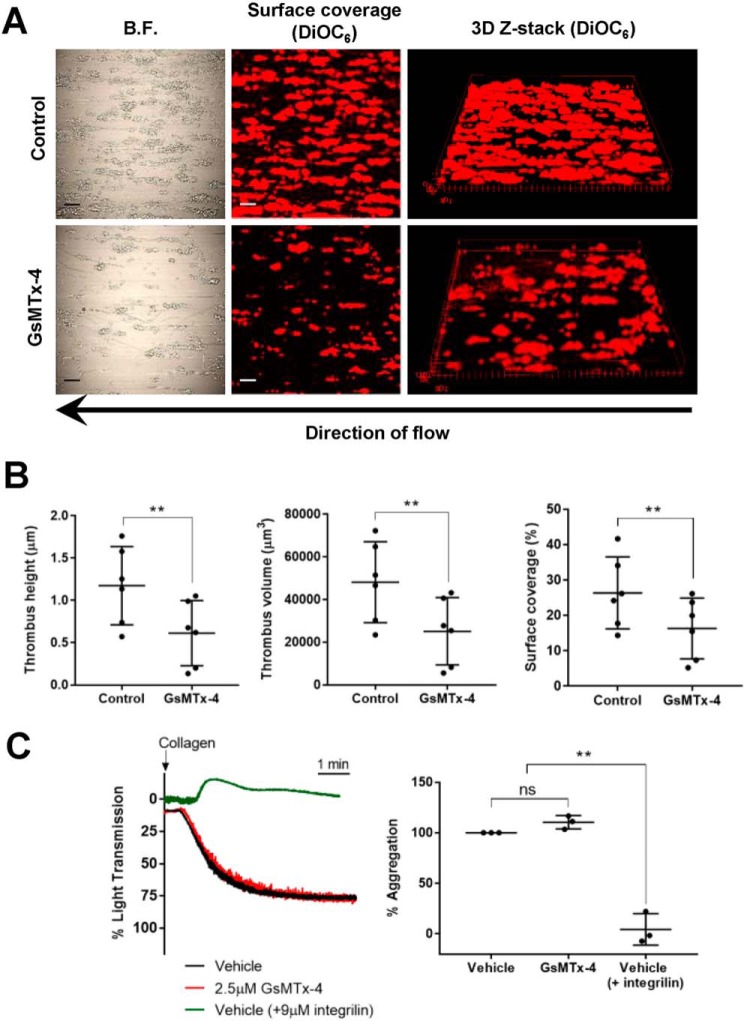
**Collagen-induced thrombus formation but not platelet aggregation is inhibited by GsMTx-4.** *A*, representative images of surface coverage and 3D *Z*-stacks for thrombi formed by DiOC_6_-stained platelets on a collagen surface under control and GsMTx-4-pretreated conditions. *Scale bars*, 20 μm. *B.F.*, bright field. *B*, average values (*n* = 6) for thrombus height, thrombus volume, and surface coverage under control and GsMTx-4-treated conditions. *C*, collagen-evoked aggregation under control and GsMTx-4-treated conditions. Integrilin treatment was performed as a control to demonstrate that aggregation is abolished by inhibition of the α_IIb_β_3_ integrin. Representative light transmission traces are shown in the *left panel*, and average maximal light transmission responses expressed as percentages of aggregation are shown in the *right panel* (*n* = 3). **, *p* < 0.01. *ns*, not significant.

Pretreating whole blood with GsMTx-4 for 30 s consistently resulted in a marked reduction in thrombus formation under arterial flow on a collagen surface (Fig. 3*A*). All three aspects of thrombus dimension analyzed (height, volume, and surface coverage) were reduced compared with control conditions; mean thrombus height to 52% of control (1.2 ± 0.5 to 0.6 ± 0.4 μm), mean thrombus volume to 52% of control (48,133 ± 18,957 to 25,126 ± 15,759 μm^3^), and mean surface coverage to 62% of control (26.4 ± 10.2 to 16.3 ± 8.6%) (Fig. 3*B*). In contrast, GsMTx-4 had no effect on the aggregation response of platelets to 1 μg/ml collagen measured in stirred suspensions, which induce minimal levels of shear (Fig. 3*C*). This response depends upon activation of the α_IIb_β_3_ integrin (27), as demonstrated by the effect of the inhibitor integrilin (Fig. 3*C*). Together, these data suggest that the underlying GsMTx-4-sensitive pathway is crucially dependent upon application of shear stress for its activation.

### Effect of GsMTx-4 on previously identified Ca^2+^ entry pathways of human platelets

**Figure 4.**
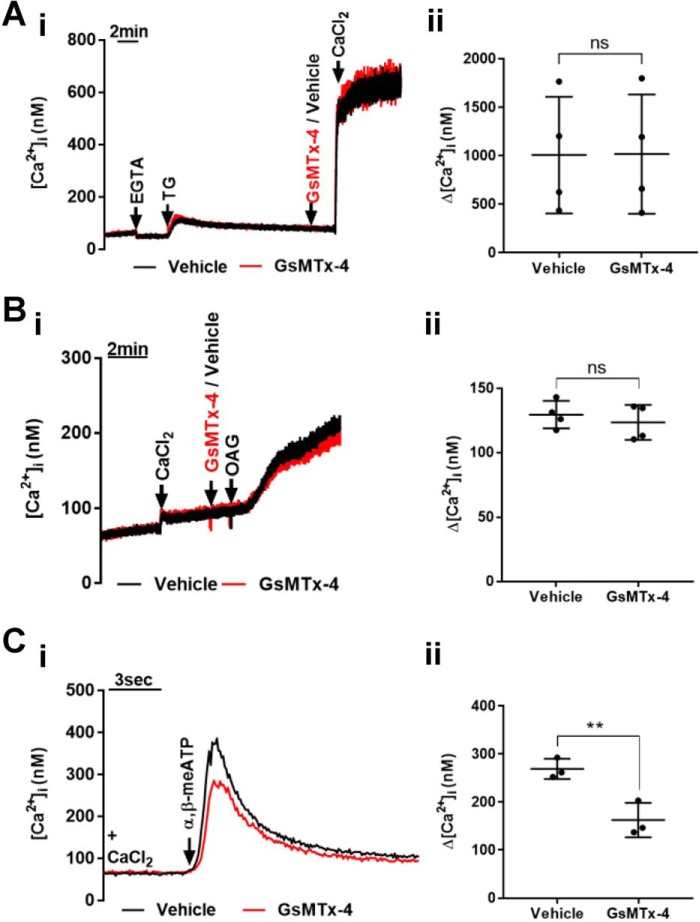
**Effect of GsMTx-4 on Ca^2+^ entry via TRPC6, P2X1, and store-operated channels in platelets.** *A–C*, representative [Ca^2+^]*_i_* recordings (*left panels*) and average peak [Ca^2+^]*_i_* responses (*right panels*) for store-operated (*n* = 4) (*A*), TRPC6 (*n* = 4) (*B*), and P2X1 cation channels (*n* = 3) (*C*) in suspensions of platelets in the presence and absence of GsMTx-4. Store-operated Ca^2+^ entry was assessed by addition of 1.26 mm CaCl_2_ 15 min after treatment with the SERCA inhibitor thapsigargin. TRPC6 was activated using the diacylglycerol analogue OAG. P2X1 was activated with the non-hydrolyzable ATP analogue α,β-meATP (10 μm). **, *p* < 0.01; *ns*, not significant.

**Figure 5.**
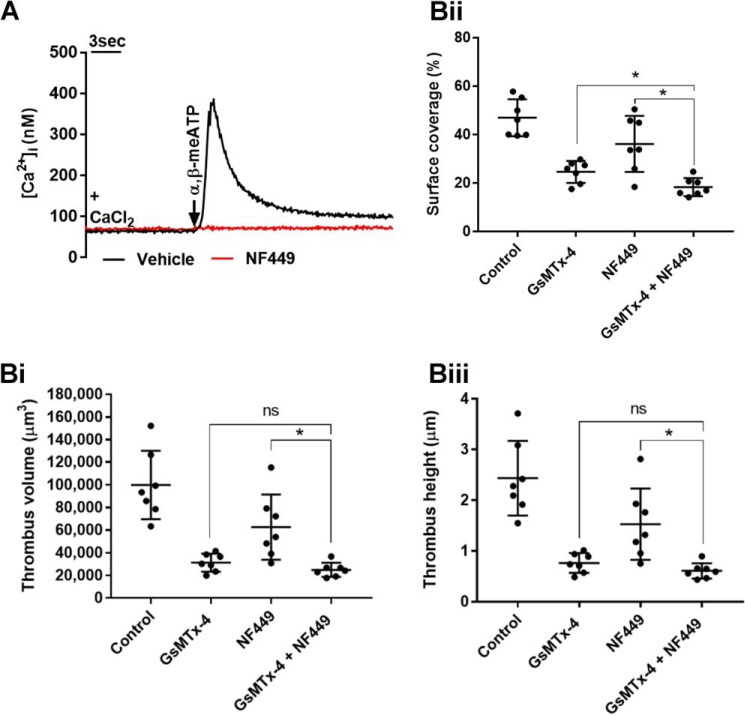
**GsMTx-4 inhibits thrombus formation independently of P2X1 receptors.** *A*, P2X1-dependent Ca^2+^ entry (α,β-meATP, 10 μm) in platelet suspensions is completely inhibited by 1 μm NF449. *B*, effect of 2.5 μm GsMTx-4 and 1 μm NF449, individually and combined, on thrombi formed on a collagen surface. The average values are shown (*n* = 7) for thrombus volume (*panel i*), surface coverage (*panel ii*), and thrombus height (*panel iii*). *, *p* < 0.05. *ns*, not significant.

GsMTx-4 is widely used as an inhibitor of MS ion channels; however, its effects on well established platelet Ca^2+^ entry pathways are unknown. Store-operated Ca^2+^ entry through Orai1 channels is a major Ca^2+^ entry pathway evoked by multiple agonists and can be selectively activated by depletion of intracellular Ca^2+^ stores with the SERCA inhibitor thapsigargin (3, 4). GsMTx-4 had no significant effect on store-operated Ca^2+^ entry assessed from the peak increase in Ca^2+^ after addition of 1.26 mm Ca^2+^ to platelets pretreated with 1 μm thapsigargin for 15 min in nominally Ca^2+^-free saline (Fig. 4*A*, *panels i* and *ii*). Furthermore, GsMTx-4 caused no inhibition of Ca^2+^ entry through transient receptor potential cation channel subfamily C member 6 (TRPC6) ion channels directly activated using the diacylglycerol analogue 1-oleoyl-2-acetyl-glycerol (OAG) (60 μm) (28) (Fig. 4*B*, *panels i* and *ii*). A third Ca^2+^-permeable pathway in platelets is the ATP-gated P2X1 channel, which can be selectively activated by α,β-meATP (29). Interestingly, a 30-s pretreatment with GsMTx-4 led to a reduction in the peak Ca^2+^ response (to 60% of control) to a supramaximal concentration of α,β-meATP (10 μm; a decrease from 296.1 ± 21.1 to 162.4 ± 35.9 nm) (Fig. 4*C*, *panels i* and *ii*). Loss of P2X1 receptor activity did not contribute to the inhibition of platelet shear-dependent Ca^2+^ responses by GsMTx-4 (Fig. 2) because these experiments were carried out in the absence of apyrase, which leads to complete desensitization of these ATP-gated cation channels (29) (see Fig. 8*A*). However, P2X1 receptors will be functional in the thrombus formation experiments using whole blood because of the ectonucleotidase activity of plasma and leukocytes (30, 31). We therefore compared the effect of GsMTx-4 and specific inhibition of P2X1 using 1 μm NF449 on thrombus formation. This concentration of NF449, which abolishes P2X1 activity (Fig. 5A), caused a reduction in thrombus volume to 63% of control (Fig. 5*B*, *panel i*), consistent with previous reports (32). Importantly, the combined addition of GsMTx-4 and NF449 caused a more significant inhibition of thrombus formation compared with NF449 alone (Fig. 5*B*). Using NF449 alone, mean thrombus volume was reduced from 99,856 ± 30,184 μm^3^ to 62,678 ± 28,802 μm^3^(63% of control), whereas using GsMTx-4 alone and GsMTx-4 and NF449 combined, there were reductions to 31% (to 31,290 ± 7986 μm^3^) and 25% (to 24,889 ± 6251 μm^3^) of control in thrombus volume, respectively (Fig. 5*B*, *panel i*). Similarly, mean percentage surface coverage was reduced from 46.9 ± 7.6 to 36.1 ± 11.6% using NF449 and to 24.6 ± 4.5 and 18.4 ± 3.7% using GsMTx-4 and both GsMTx-4 and NF449, respectively (Fig. 5*B*, *panel ii*). Mean thrombus height decreased from 2.4 ± 0.7 to 1.5 ± 0.7 μm using NF449 and to 0.8 ± 0.2 and 0.6 ± 0.2 μm using GsMTx-4 and both GsMTx-4 and NF449, respectively (Fig. 5*B*, *panel iii*). Together, these results indicate that a pathway other than P2X1 receptors is the main target for GsMTx-4 during inhibition of thrombus formation.

### MS cation channel expression in platelets

**Figure 6.**
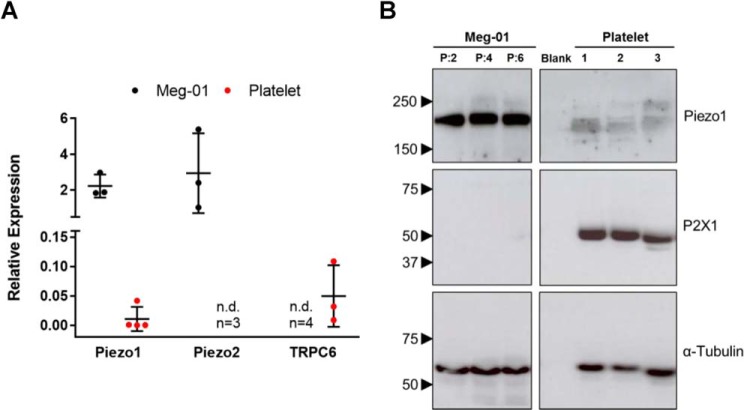
**Mechanosensitive ion channel expression in human platelets and the Meg-01 cell line.** *A*, relative expression of mRNA transcripts for three MS cation channels (Piezo1, Piezo2, and TRPC6) in human platelets and the Meg-01 cell line, relative to GAPDH. *n.d.*, not detected. The values are shown only for detectable levels of expression. *B*, Western blots for Piezo1 (233 kDa) and P2X1 receptors (55 kDa) in Meg-01 and human platelet lysates, compared with α-tubulin housekeeping control (60 kDa). The sizes (in kDa) and positions of protein standards are indicated with *arrowheads*. Meg-01 samples were from three different culture passages (*lanes P:2*, *P:4*, and *P:6*), and platelet samples were from three different donors (*lanes 1*, *2*, and *3*). The *blank lane* lacked protein lysate.

Within a transcriptomic screen of human platelets for all known ion channels, Piezo1 and TRPC6 were the only MS cation channels detected (5). Piezo1 (*FAM38A*) was detected at trace levels in this recent study; thus we repeated the qPCR assay using a larger sample volume. Parallel qPCR runs with primers for GYPA and CD45 were used as described previously (5) to ensure that platelet samples were free from contamination by erythrocytes and leukocytes. In these purified platelet samples, we detected quantifiable levels of *FAM38A* but not the related family member *FAM38B*, encoding Piezo2 (Fig. 6*A*). Meg-01 cells were found to express higher levels of *FAM38A* transcripts compared with platelets and to also express *FAM38B* (Fig. 6*A*). In contrast, TRPC6 mRNA was not detected in Meg-01 cells, although it was present in platelets as reported previously (28). Piezo1 protein was also detected using Western blotting in both cell types (Fig. 6*B*), and this further suggested a lower level of expression in platelets compared with Meg-01. Piezo1 protein in humans is known to be an *N*-linked glycoprotein (33, 34), and the diffuse nature of the band obtained for Piezo1 may result from heterogeneity in glycosylation as reported in the immunoblots of other glycoproteins (35). The opposite order of expression was observed for P2X1 protein in the two cell types, which was included as a positive control ion channel target in platelet lysates. Lack of P2X1 expression in Meg-01 cells was also confirmed by ratiometric [Ca^2+^]*_i_* measurements where no Ca^2+^ responses were obtained following treatment with a supramaximal concentration of the P2X1 agonist, α,β-meATP, in the presence of apyrase (Fig. 8*B*). Although three alternatively spliced forms of Piezo2 protein were detected in Meg-01 cells, they were all absent in platelets,^4^ in agreement with the lack of Piezo2 mRNA (Fig. 6*A*). Piezo1 channels have been previously studied in various tissues including erythrocytes with the aid of the blocker GsMTx-4 (5, 28, 36) and thus could represent the GsMTx-4-sensitive shear-induced Ca^2+^ entry we observe in Meg-01 cells and platelets.

### The Piezo1 agonist Yoda1 induces Ca^2+^ influx in platelets

**Figure 7.**
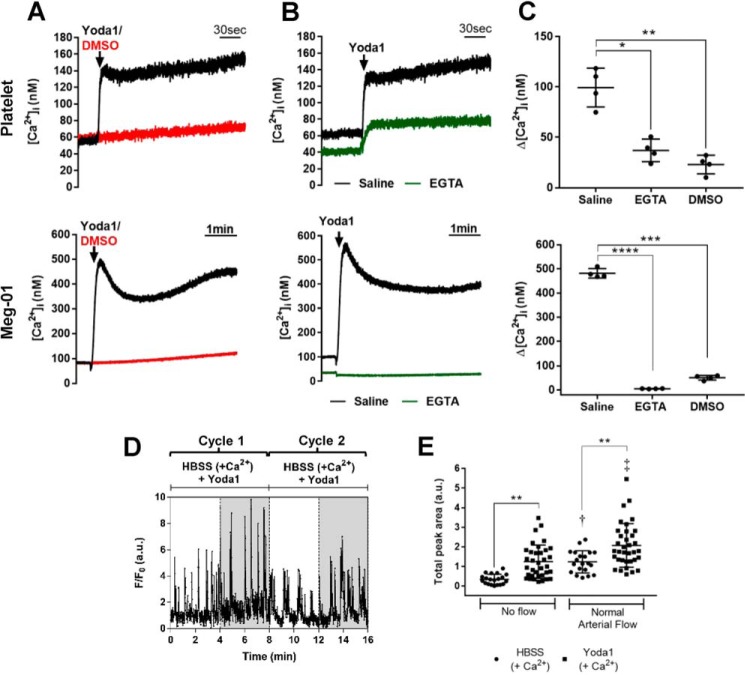
**The Piezo1 agonist Yoda1 induced increases in [Ca^2+^]*_i_* in platelets and Meg-01 cells.** *A–C*, [Ca^2+^]*_i_* responses to Yoda1 (25 μm) assessed in stirred Fura-2-loaded washed suspensions of platelets (*top panels*) and Meg-01 cells (*bottom panels*). *A* and *B* show representative recordings, and *C* shows the average peak [Ca^2+^]*_i_* increases (*n* = 4) for Yoda1 in the presence of extracellular Ca^2+^ compared with its vehicle control (DMSO) and following removal of external Ca^2+^ (EGTA). *D*, representative intracellular Ca^2+^ recording (Fluo-3 *F*/*F*_0_ fluorescence) from a single platelet attached to a PECAM-1-coated glass coverslip in the presence of Yoda1 and exposed to two cycles of no flow (*white regions*) and arterial shear (*gray regions*). See Fig. 2*C*, (*panel i*) for the control trace. *E*, average Ca^2+^ increases above baseline (*F*/*F*_0_·4 min) in the presence and absence of Yoda1 under conditions of no flow and normal arterial shear (*n* = 20, 35, 20, and 35 cells in HBSS no flow, Yoda1 no flow, HBSS normal arterial, and Yoda1 normal arterial conditions, respectively). **, *p* < 0.01; †, *p* < 0.01 compared with no flow, in the presence of HBSS; ‡, *p* < 0.001 compared with no flow in presence of Yoda1. Apyrase was omitted from the extracellular buffer to avoid P2X1 receptor responses (see Fig. 8).

**Figure 8.**
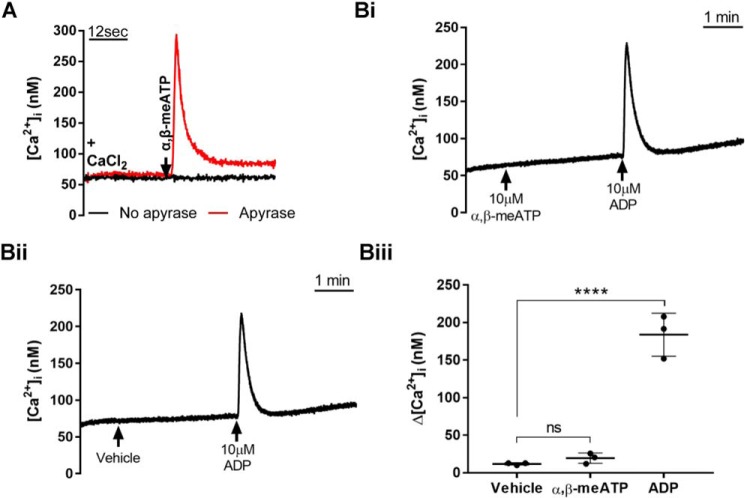
**Assessment of P2X1 activity in Fura-2-loaded platelet and Meg-01 cell suspensions in the presence and absence of extracellular apyrase.** *A*, selective activation of platelet P2X1 channels using α,β-meATP in the presence and absence of 0.32 unit/ml apyrase. *B*, representative [Ca^2+^]*_i_* recordings (*panels i* and *ii*) and average Ca^2+^ increases (*panel iii*) demonstrating that α,β-meATP does not induce [Ca^2+^]*_i_* elevations in Meg-01 cells similar to vehicle control, indicating no P2X1 activity in these cells. As a positive control, the addition of a supramaximal concentration of ADP is shown to cause sharp [Ca^2+^]*_i_* elevations indicating intact P2Y responses. ****, *p* < 0.0001. *ns*, not significant.

To further assess the contribution of Piezo1 channels to platelet signaling and functional events, we used the recently characterized Piezo1 activator, Yoda1 (15). In Fura-2 ratiometric measurements from stirred suspensions, Yoda1 caused a substantial, immediate, and sustained elevation of [Ca^2+^]*_i_* in both platelets and Meg-01 cells when Ca^2+^ was present in the extracellular milieu (Fig. 7*A*). Most or all of this response was lost in Ca^2+^-free external saline (decrease to 37% of control, from 99.4 ± 19.3 to 37.0 ± 11.1 nm in platelets; and to 1% of control, from 481.3 ± 19.6 to 5.2 ± 0.9 nm in Meg-01), as expected if the predominant location of Piezo1 channels is on the surface membrane (Fig. 7, *B* and *C*). The residual response to Yoda1 in platelets in Ca^2+^-free medium can be explained by the suggested presence of Piezo1 channels on membranes of the intracellular stores (15). These experiments were conducted in apyrase-free medium to abolish P2X1 receptor activity, which was confirmed by the absence of responses to α,β-meATP (Fig. 8*A*). Yoda1 also enhanced intracellular Ca^2+^ transients in platelets attached to slides via PECAM-1 (Fig. 7, *D* and *E*). Yoda1 increased the occurrence of Ca^2+^ transients both in the absence of flow and upon application of shear (compare Fig. 7*D* with Fig. 2*C*, *panel i*). Under static conditions, the *F*/*F*_0_ integral increased more than 3-fold from a *F*/*F*_0_·4 min value of 0.3 ± 0.3 to 1.2 ± 0.9. Under normal arterial flow, in the absence of Yoda1 the *F*/*F*_0_·4 min value was 1.2 ± 0.6, which showed a 1.7-fold increase to 2.1 ± 1.1 in the presence of the Piezo1 agonist (Fig. 7*E*).

## Discussion

Since their identification in 2010 by the Patapoutian group (18), the MS Piezo ion channels have received considerable attention and been shown to contribute to functional responses across a range of cell types (10, 12, 14). Furthermore, disease conditions have been reported that result from both gain-of-function and loss-of-function mutations of these cation-permeable channels (16, 36, 37). Of relevance to the present work, Piezo1 channels play crucial mechanotransduction roles in the cardiovascular system, particularly in red blood cells and endothelial cells where they regulate cell volume homeostasis (10) and vascular development (12), respectively. In the circulation, the mechanical forces of shear have a well established influence on platelet activation (38). For example, the ability of von Willebrand factor to engage its receptors on the platelet surface is enhanced by increased shear (39). In addition, platelet membrane morphological events respond to physical influences in a PI3K-dependent manner (40). However, mechanisms for more direct mechanical activation of platelet signaling events have not been identified (38).

Several pieces of evidence support the conclusion that platelets possess MS Ca^2+^ influx mechanisms that are activated by flow, and thus shear forces, and that the underlying pathway is Piezo1. First, expression of mRNA transcripts and protein for Piezo1 was detected in both platelets and Meg-01 cells (5) (Fig. 6), but the Piezo2 protein product was not detected in platelets,^4^ in agreement with previous proteomic studies (7). Second, individual platelets and Meg-01 cells that were attached to a surface without causing activation, displayed [Ca^2+^]*_i_* responses upon exposure to arterial levels of shear that were inhibited by the MS channel blocker GsMTx-4 or removal of external Ca^2+^. Third, Yoda1, a recently described chemical agonist of Piezo1 that does not activate Piezo2 (10, 15), directly stimulated Ca^2+^ entry into both platelets and Meg-01 cells in the absence of shear and also potentiated shear-dependent Ca^2+^ transients in platelets. In agreement with these data, Patapoutian and co-workers (15) demonstrated that Yoda1 can induce Piezo1 activation in the absence of mechanical stimulation and also increase its sensitivity to mechanical activation. Although GsMTx-4 is a general MS ion channel blocker, it has been the main inhibitor used in the study of Piezo1 function (11, 13). The toxin acts through insertion into the lipid bilayer and modification of the lipid:channel interface (41) and hence may influence a number of ion channels. However, GsMTx-4 did not block TRPC6 or Orai1 store-operated Ca^2+^ entry in platelets (Fig. 4). Although P2X1 receptors were partially inhibited by GsMTx-4, these ATP-gated Ca^2+^-permeable channels will have been desensitized in the ectonucleotidase (*i.e.* apyrase)-free conditions used to record shear-induced Ca^2+^ transients in our study (29). Furthermore, in the thrombus formation assay using whole blood, which retains significant ectonucleotidase activity and thus also P2X1 receptor activity (30–32), the GsMTx-4 block of thrombus formation was still observed after abrogation of P2X1 receptor responses with NF449. It is worth noting that intravital imaging studies have recently suggested that blood rheology is the primary factor driving thrombus formation *in vivo*, with less significant roles for classical diffusible platelet agonists (42, 43). Ca^2+^ influx through Piezo1 channels certainly represents a candidate for transduction events directly influenced by rheological forces in the arterial circulation.

Although no previous report has identified a molecular mechanism for MS Ca^2+^ influx in platelets, the presence of such a pathway has been suggested in earlier studies (44, 45). Using an approach to monitor [Ca^2+^]*_i_* within a cone-and-plate viscometer, Kroll and co-workers (45) demonstrated a transmembrane Ca^2+^ influx in response to arterial or higher levels of shear. In addition, Simon and co-workers (44) report a link between transmembrane Ca^2+^ flux and hemodynamic shear stress from studies of hypertensive patients. Piezo1 could account for these previously reported shear-dependent Ca^2+^ influx pathways, yet we recognize the need for further work to address this possibility. The level of expression of Piezo1 in platelets was low; however, it was detectable both at the mRNA transcript and protein levels. By comparison, the lowest density ion channel observed within patch clamp recordings from human platelets (KCa3.1) (4, 46) was below the detectable level within a qPCR screen (5). Considering the large surface-area-to-volume ratio of the mammalian platelet, it is also worth noting that a Ca^2+^-permeable ion channel need only be present at a low copy number or exhibit a low open probability to significantly influence the intracellular Ca^2+^ concentration.

Piezo1 channels were also clearly expressed and functional in the Meg-01 human megakaryoblastic cell line, which has been used as an alternative system for studies of platelet signaling events (4, 17). This finding also raises the possibility that Piezo1 may contribute to megakaryocyte function, although further experimental evidence is needed to address this possibility. For example, shear forces are important during thrombopoiesis by promoting platelet release from proplatelet extensions within the venous sinusoids (47, 48). In addition, megakaryocytes have been postulated as active participants in the mechanosensitivity of the marrow environment that regulates the bone mass, likely through interactions with osteoblasts (49). A noticeable difference between the shear-dependent Ca^2+^ responses of Meg-01 cells and platelets was the more immediate nature of the cell line response compared with the delayed increase in platelets (Figs. 1*B* and 2*C*). A likely explanation is the very different cytoskeletal arrangement of platelets, which consists of a cortical cytoskeleton that is responsible for its discoid resting shape and also results in a less flexible plasma membrane compared with other cell types, including its precursor and related cell lines (25, 26). Because Piezo1 is gated by tensions within the lipid bilayer of the membrane itself rather than via a link to the cytoskeleton (8, 9, 18), platelets may need to undergo a greater deformation by the fluid shear compared with Meg-01 cells before channel activation. Although Piezo1 channels are gated by bilayer tension in cytoskeleton-free artificially generated blebs, Cox *et al.* (9) have emphasized that cytoskeletal proteins or links to the extracellular matrix components can modify the tension experienced by the bilayer in intact cells. This cytoskeletal “mechanoprotection” effect is known to curb the activity of endogenous Piezo channels (50, 51). Manipulating the cytoskeletal properties of cells has also been linked to changes in latency of channel activation and channel gating in general (9, 52). In our studies, a second application of increased shear stimulated Ca^2+^ transients with reduced delay, similar to effects on stretch-activated K^+^ channels in *Lymnaea* neurons, where it has been suggested that application of repeated pressure causes cytoskeleton-dependent adaptation (53).

In conclusion, we show that human platelets express a MS Ca^2+^ entry pathway that is activated by arterial shear stress *in vitro*. Piezo1 is the main candidate for the underlying MS channel mediating this effect. Pharmacological inhibition of MS channels indicates that they contribute to thrombus formation under arterial flow. However, future work should develop an animal model lacking Piezo1 specifically in platelets and megakaryocytes to further support these conclusions and to extend to *in vivo* studies. MS cation channels, at the pathologically high levels of shear stress that are generally experienced at the regions of vessel narrowing resulting from stenosis or atherosclerosis, could potentially enhance Ca^2+^ influx, which can increase the risk of life-threatening thrombus formation.

## Experimental procedures

### Materials

The MS ion channel inhibitor GsMTx-4 peptide (STG-100) was from Alomone Labs (Jerusalem, Israel), and the agonist of Piezo1, Yoda1 (5586), was from Tocris Bioscience (Bristol, UK). Fura-2 AM and Fluo-3 AM were from Invitrogen. PECAM-1 antibody (WM59) (MCA1738T) for platelet attachment was purchased from AbD Serotec (Kidlington, UK). Type I collagen (Horm, from equine tendon) was from Takeda (Linz, Austria). Unless otherwise stated, all other materials were from Sigma-Aldrich.

### Platelet and Meg-01 sample preparation

Blood was collected by venepuncture from informed, consenting healthy volunteers, in accordance with the Declaration of Helsinki. This study was approved by the University of Leicester Research Ethics Committee for Human Biology (non-National Health Service). In experiments where human platelets were used, the data were obtained from three to seven donors. Whole blood for thrombus formation was collected into 40 μm Phe-Pro-Arg-chloromethylketone (Haematologic Technologies Inc., Essex Junction, VT) as anticoagulant. For all other studies, blood was taken into acid-citrate-dextrose (ACD) solution (85 mm trisodium citrate, 78 mm citric acid, 111 mm glucose) at a ratio of 6:1 (blood:ACD). For extraction of mRNA, 1.5 ml of platelet inhibitor mixture containing 0.1 μm prostaglandin E_1_, 2 mm EDTA, and 0.3 mm acetylsalicylic acid in ACD was added per 10 ml of whole blood or Meg-01 cell suspension. Platelet-rich plasma (PRP) was prepared by centrifugation at 150 × *g* for 20 min for mRNA extraction, 100 × *g* for 20 min for aggregometry, or 700 × *g* for 5 min for other experiments. For preparation of washed platelets (WP) for aggregometry, ACD was added to PRP in 1:80 (ACD:PRP) ratio and 1 μm prostaglandin E_1_ and then centrifuged at 700 × *g* for 10 min, and the platelets were resuspended in Tyrode's HEPES buffer (134 mm NaCl, 2.9 mm KCl, 0.34 mm Na_2_HPO_4_, 12 mm NaHCO_3_, 20 mm HEPES, 0.84 mm MgCl_2_, 10 mm glucose), after which they were centrifuged at 700 × *g* for 10 min. WP were resuspended in a volume of Tyrode's HEPES buffer equivalent to that of the original PRP. For intracellular Ca^2+^ measurements, the PRP was incubated at room temperature for 15 min with 100 μm acetylsalicyclic acid and 0.32 unit/ml apyrase (type VII; Sigma) and then incubated with either 2 μm Fura-2 AM (45 min, 37 °C) or 5 μm Fluo-3 AM (45 min, room temperature). The PRP was then centrifuged at 350 × *g* for 20 min, and the platelets were resuspended in an equal volume of nominally Ca^2+^-free saline (145 mm NaCl, 5 mm KCl, 1 mm MgCl_2_, 10 mm HEPES, 10 mm glucose, pH 7.35) that also contained 0.32 unit/ml apyrase for experiments when P2X1 receptor responses were being studied. Immediately prior to Fura-2 measurements in cuvettes or before introducing Fluo-3-loaded platelets to biochips for attachment, the platelet suspension was diluted 1:1 with Ca^2+^-free saline. For platelet lysate preparation for Western blotting, the protocol for Fura-2-loaded platelet preparation was followed without the dye addition step, and the washed platelet suspension as lysed with radioimmunoprecipitation assay buffer (150 mm NaCl, 50 mm Trizma hydrochloride, 0.5% sodium deoxycholate, 0.1% SDS, 1% Triton X-100) with EDTA-free protease inhibitor mixture (cOmplete mini; Roche) on ice for 1 h.

Meg-01 cells were obtained from the European Collection of Authenticated Cell Cultures and grown in RPMI 1640 supplemented with 10% FBS and 100 units/ml penicillin-streptomycin. Sterilized glass coverslips were coated with 0.1 mg/ml poly-d-lysine hydrobromide (molecular weight > 300,000) for 15 min. The slides were then rinsed with sterile water and dried for 2 h. Meg-01 cells in medium were added to the coated slides and incubated at 37 °C for 15 min to promote attachment. Attached cells were then treated with 2 μm Fluo-3 AM at room temperature for 45 min. After washing in Hanks' balanced salt solution (HBSS; 5.33 mm KCl, 0.44 mm KH_2_PO_4_, 137.93 mm NaCl, 4.17 mm NaHCO_3_, 0.34 mm Na_2_HPO_4_, 5.56 mm d-glucose, 5.00 mm HEPES, 0.49 mm MgCl_2_, 0.41 mm MgSO_4_, and 1.26 mm CaCl_2_), slides were immediately inserted in a parallel-plate flow chamber for experimentation. Meg-01 cell lysates were prepared by washing with PBS and lysed as described for platelets. The lysates were centrifuged at 20,000 × *g* at 4 °C for 10 min, and the supernatant was stored at −80 °C. Meg-01 cells used in all experiments were from passage numbers 1–14.

### Fluorescence imaging

**Figure 9.**
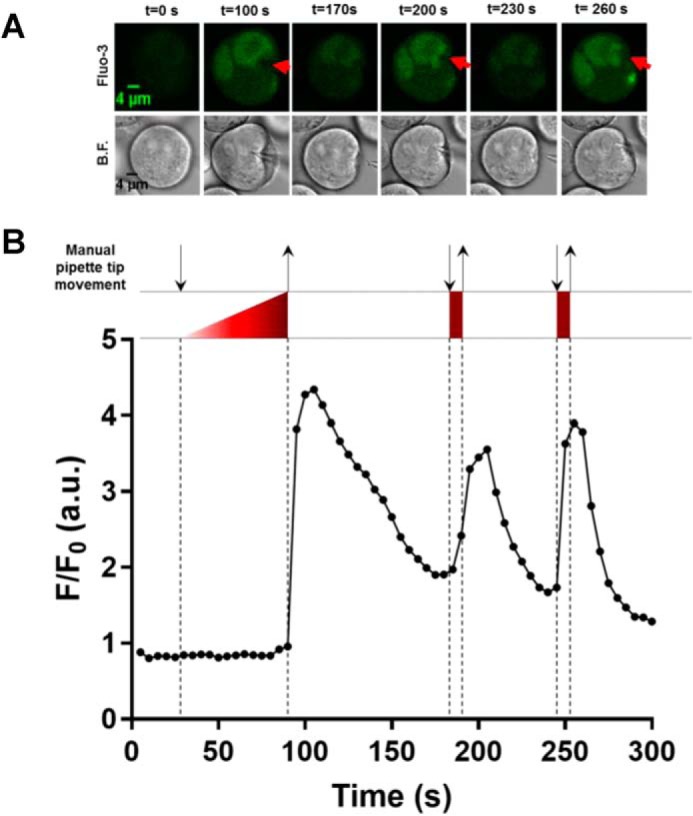
**Mechanical stimulation of Meg-01 cells with a glass pipette tip results in [Ca^2+^]*_i_* elevations.** *A*, representative images of a Fluo-3-loaded Meg-01 cell at specified time points before and during [Ca^2+^]*_i_* elevations stimulated by depression of the plasma membrane with a blunt-ended glass micropipette. The extracellular saline (HBSS) contained 1.26 mm Ca^2+^. The *red arrowheads* indicate the positions and the directions in which the glass probe was applied. Similar responses were obtained from 14 Meg-01 cells from three different cultures. *B.F.*, bright field. *B*, the *F*/*F*_0_ fluorescence recording of the Meg-01 cell shown in *A*. The *downward arrows* indicate when a push was applied onto the cell, and *upward arrows* indicate release of push. The regions enclosed with *dashed lines* represent the duration of a mechanical push by the glass probe.

Imaging of thrombus formation and [Ca^2+^]*_i_* recordings from single platelets or Meg-01 cells was carried out on an Olympus IX81 inverted confocal microscope with a FV1000 laser scanning module (Olympus, UK) using a 60×, 1.35 NA oil immersion lens (UPLSAPO). The confocal aperture was set for optimal optical slicing (1 Airy unit, slice thickness ≈1.25 μm). Fluo-3 fluorescence images were captured at a rate of 1.74 Hz for platelets and 0.37 Hz for Meg-01 cells (Figs. 1, 2, 7, and 9).

### Thrombus formation under flow

The glass coverslips were coated with collagen (100 μg/ml) overnight in a humidified chamber at 4 °C. Whole blood was stained with 1 μm DiOC_6_ on a rotor at room temperature for 30 min before use. A programmable AL-1000 syringe pump (World Precision Instruments, Sarasota, FL) attached to a parallel-plate flow chamber was used to initially introduce HEPES-buffered saline solution (150 mm NaCl, 5 mm KCl, 1 mm MgSO_4_, 10 mm HEPES) to remove air bubbles. 1 ml of whole blood was then introduced into the system for a period of 5 min at the required shear level before perfusing HEPES-buffered saline to clear the components unbound to the collagen surface. The blood was applied at a flow rate of 0.235 ml/min, which equals to a normal arterial shear rate of 1002.6 s^−1^, calculated according to Equation 1,

(Eq. 1) $$Q=\frac{wh^{2}t}{6\mu }$$

where *Q* = flow rate (ml/s), *w* = microslide lumen width in cm (0.15), *h* = microslide lumen height in cm (0.0125), *t* = shear stress (Pa), and μ = viscosity of whole blood (0.001002 Pa/s). To calculate shear rate (s^−1^), *t* was divided by μ.

Time-series scans were performed during thrombus formation, and subsequent *Z*-stack analyses of stable thrombi were carried out within 15 min of their formation using step changes (Δ*Z*) of 0.69 μm. The data represent averages of at least four randomly chosen fields per experiment. Analysis and 3D reconstruction of *Z*-stacks were performed with ImageJ 1.49 Volume Viewer 2.0 plugin (National Institutes of Health). Surface coverage and thrombus volume were calculated according to the Cavalieri principle as previously described (54, 55). The heights of the thrombi were calculated by dividing the total thrombus volume by the area of the field.

### Ca^2+^ imaging in Meg-01 cells and human platelets under flow

Fluo-3-loaded Meg-01 cells attached to slides were treated with 2.5 μm GsMTx-4 for 1 min or vehicle (HBSS) as necessary before inserting the slides into the parallel-plate flow chamber. To apply fluid shear stress, the reservoir was filled with either HBSS, Ca^2+^-free HBSS containing 1 mm EGTA or HBSS containing 2.5 μm GsMTx-4 as appropriate, and the AL-1000 syringe pump (World Precision Instruments) was set to draw fluid through the system at the shear rates of 1002.6, 2282.67, and 3989.248 s^−1^, which represent shear conditions in normal arteries, moderately stenotic arteries, and severely stenotic arteries, respectively (56). The fluorescent signals were background-corrected, and fluorescence levels (*F*) were normalized against prestimulus fluorescence level (*F*_0_) to yield *F*/*F*_0_ values.

### Imaging of Ca^2+^ transients in single platelets

Platelets were loaded with Fluo-3 (5 μm Fluo-3-AM for 45 min, at room temperature) and attached onto glass-bottomed Vena8 GCS biochips (Cellix Ltd., Dublin, Ireland) that were coated with monoclonal mouse anti-human PECAM-1 (CD31) antibody by incubation at 37 °C for 1 h. Excess antibody was removed, and nonspecific sites were blocked with 2% BSA at 37 °C for 1 h to prevent glass-induced platelet activation. Immediately prior to each experiment, Fluo-3-loaded platelets were introduced into the biochip channel and incubated at 37 °C for 10 min with occasional gentle shaking. The biochip was then mounted on the microscope stage, and HBSS was introduced into the channel under gravity. Pharmacological reagents (2.5 μm GsMTx-4 or 25 μm Yoda1) were introduced at a very low shear rate (410.16 s^−1^) prior to the experimental recording. Captured time-series images were analyzed on ImageJ, version 1.47 (57) using the Time Series Analyzer V2.0 plugin to obtain the Ca^2+^ traces. The *F*/*F*_0_ values were calculated after background and *F*_0_ correction, and values were transferred to GraphPad Prism 6 software for quantification of the *F*/*F*_0_ integral over 4 min (*F*/*F*_0_·4 min, in arbitrary units). The baseline was set manually, and peaks more than 1 point above baseline were included within the calculation of a Ca^2+^ increase.

### Light transmission aggregometry

Platelet aggregometry was performed in a Chronolog 400 lumi-aggregometer (Chrono-Log Corporation, Havertown, PA), at 37 °C. Platelets were resuspended in nominally Ca^2+^-free saline, to which 2 mm CaCl_2_ and 100 μg/ml fibrinogen were added at the start of each experiment. WP were incubated with 2.5 μm GsMTx-4 or vehicle for 30 s, respectively, before the addition of collagen to stimulate aggregation. Where necessary, aggregation was inhibited by the inclusion of 9 μm integrilin 3 min before stimulation with collagen. The percentage of light transmission was converted to the percentage of aggregation by normalizing the vehicle light transmission response to 100% aggregation.

### Fura-2 ratiometric Ca^2+^ measurements

Fura-2 ratiometric Ca^2+^ measurements were performed in stirred suspensions using a Cairn spectrofluorimeter system (Cairn Research Ltd., Faversham, UK) at 37 °C as described elsewhere (29). After loading with Fura-2, the cells were resuspended in nominally Ca^2+^-free saline, and where necessary, 2 mm CaCl_2_ was added to individual cuvettes 30s before experimental treatments. Fura-2 fluorescence was converted to [Ca^2+^]*_i_* (nm) using an extracellular calibration after digitonin permeabilization (29). Peak [Ca^2+^]*_i_* responses (*i.e.* Δ[Ca^2+^]*_i_* values) represent the increases in [Ca^2+^]*_i_* above the prestimulus concentration. The materials used to study Ca^2+^ influx were as follows: 0.2 mm EGTA, 1 μm thapsigargin to induce store depletion (58); 60 μm OAG, a cell-permeable diacylglycerol derivative and TRPC6 channel agonist (59, 60); and 10 μm α,β-methylene ATP (α,β-meATP), to selectively activate P2X1 receptors.

### mRNA extraction and quantitative real-time polymerase chain reaction

mRNA extraction from platelets and Meg-01 cells, and quantitative PCR analysis were performed using QuantiTect Primer assays (Qiagen), as previously described (5). The reaction end products were run on a 1.5% agarose gel to confirm specificity of the primers.^4^ mRNA expression values relative to GAPDH were calculated as previously described (61).

### Western blot analysis

Western blotting was performed as described previously (62). Briefly, 20 μg of protein sample was loaded in each well and run in a 7% acrylamide gel, and visualization of bands was achieved using Amersham Biosciences ECL Prime detection kit (GE Healthcare). The antibody concentrations used were: *FAM38A* (Piezo1) rabbit anti-human polyclonal antibody (15939-1-AP; ProteinTech, Manchester, UK), 1:2000; P2X1 rabbit anti-human polyclonal antibody (APR-001; Alomone Labs, Israel), 1:1000; and *FAM38B* (Piezo2) rabbit anti-human polyclonal antibody (G-20 Santa Cruz Biotechnology, Heidelberg, Germany), 1:200. The specificity of the antibodies for their intended target in Western blots (Fig. 6) has been previously validated using knockdown of endogenous expression, heterologous expression, or tissue from receptor-deficient mice (63–68). The widely used α-tubulin mouse anti-human monoclonal antibody (CP06; Calbiochem), 1:1000 was used as a control.

### Data analysis

All statistical analyses were performed using GraphPad Prism 6.0 software or Origin 2015 Sr2 for Windows (La Jolla, CA). One-way analysis of variance followed by Tukey's post hoc multiple comparison analyses or paired two-tailed Student's *t* tests were performed as appropriate. All results are shown as the means ± S.D. The *p* values <0.05 were considered statistically significant.

## Author contributions

Z. I. designed, performed, and analyzed the experiments, prepared the figures and wrote the manuscript. J. R. W. devised the mRNA extraction and quantitative-PCR protocols and generated the initial transcriptome database leading up to this project. J. M. G. and C. I. J. developed the single-platelet attachment approach using the PECAM-1 antibody that allowed the imaging of Ca^2+^ transients. A. H. G. contributed to the preparation of the manuscript, and M. P. M.-S. conceived and coordinated the study and wrote the manuscript. All authors approved the final version of the manuscript.

## Footnotes

4 Z. Ilkan, unpublished observations.

## Footnotes

3 The abbreviations used are:

GsMTx-4: *Grammostola spatulata* mechanotoxin 4
MS: mechanosensitive
PECAM-1: platelet endothelial cell adhesion molecule-1
HBSS: Hanks' balanced salt solution
TRPC6: transient receptor potential cation channel subfamily C member 6
OAG: 1-oleoyl-2-acetyl-*sn*-glycerol
SERCA: sarcoplasmic/endoplasmic reticulum Ca^2+^-ATPase
α,β-meATP: α,β-methylene ATP
qPCR: quantitative PCR
ACD: acid-citrate-dextrose
PRP: platelet-rich plasma
WP: washed platelets.
